# Case report: The safety of laparoscopic surgery for the retroperitoneal bronchogenic cyst

**DOI:** 10.3389/fonc.2022.1011076

**Published:** 2022-10-12

**Authors:** Hancong Li, Jun Xu, Qingbo Feng, Zhaolun Cai, Jiaxin Li

**Affiliations:** ^1^West China School of Medicine, West China Hospital, Sichuan University, Chengdu, China; ^2^Department of Pancreatic Surgery, West China Hospital, Sichuan University, Chengdu, China; ^3^Department of Minimal Invasive Surgery, Shangjin Nanfu Hosptial, Chengdu, China; ^4^Department of Liver Surgery and Liver Transplantation Centre, West China Hospital, Sichuan University, Chengdu, China; ^5^Department of Gastrointestinal Surgery, West China Hospital, Sichuan University, Chengdu, China

**Keywords:** bronchogenic cysts, laparoscopic surgery, retroperitoneal neoplasm, case report, retroperitoneal bronchogenic cyst

## Abstract

**Introduction:**

Bronchogenic cyst is a congenital aberration of bronchopulmonary malformation with bronchial-type, pseudostratified cylindrical epithelium. They are usually discovered in the mediastinum and intrapulmonary but are rarely encountered in retroperitoneum. We report a case of the retroperitoneal bronchogenic cyst and perform a literature review to summarize the safety of laparoscopic resection for this rare disease.

**Case presentation:**

We report a 57-year-old woman who was admitted to our hospital with no clinical symptoms and was found by chance to have masses in the adrenal gland area during a routine physical examination. An abdominal CT examination revealed a cystic lesion was found in the left suprarenal region. Afterward, the patient underwent a laparoscopic exploration. Histopathological findings confirmed the diagnosis of a retroperitoneal bronchogenic cyst. The patient recovered uneventfully without signs of recurrence during a 1-year follow-up period.

**Conclusion:**

Bronchogenic cyst is rare in the retroperitoneal region. It should be considered as one of the differential diagnoses of a retroperitoneal neoplasm, especially in the left retroperitoneal region. Laparoscopic surgery is technically feasible and safe for the treatment of patients with a retroperitoneal bronchogenic cyst.

## Introduction

Bronchogenic cysts (BCs) arise from abnormal budding of the foregut during embryogenesis, which is a benign congenital aberration of bronchopulmonary foregut malformation (1). It is typically located within the mediastinum and pulmonary parenchyma. The retroperitoneum is rarely involved (2). Particularly, retroperitoneal bronchogenic cysts(RBCs) tend to occur on the pancreas corpus or left adrenal gland (3). In most cases, BCs are asymptomatic unless they are infected, ruptured into the surrounding cavities, or enlarged enough to compress adjacent structures (4, 5). Cough, fever, pain, and dyspnea are among the most common manifestations (6). Due to the lack of characteristic clinical features, RBCs are often accidentally identified and diagnosed by imaging examinations, such as computed tomography(CT) and magnetic resonance imaging (MRI) (7). However, due to their rarity, location, variable cystic content, and non-specific imaging, they are frequently misinterpreted as cystic teratomas, adrenal tumors, or other benign and malignant retroperitoneal lesions (7–9). Although benign lesions, surgical resection is recommended to establish the diagnosis, alleviate symptoms, and prevent complications or malignant transformation (1, 10). Recently, laparoscopic procedure for RBCs has become increasingly popular for decreased scarring, less complications, shorter hospital stays, and faster recovery. Herein, we present a case of RBC, which was successfully removed through a laparoscopic excision. Additionally, we have performed a literature review to summarize the safety of laparoscopic resection for this rare disease.

## Case presentation

On January 4, 2021, a 57-year-old woman was referred to the West China Hospital for evaluation of a left adrenal neoplasm suspected of being an adrenal tumor. The patient had no symptoms and the mass was incidentally discovered on medical examination. There were no obvious abnormalities in her medical history or physical examination. Additionally, she did not have any underlying diseases or take any medications. Routine laboratory investigations such as complete blood counts and liver and kidney function tests were within normal ranges. Specifically, negative results were obtained for all adrenal gland hormones and serum tumor markers (**Table 1**).

**Table:** Table 1 Laboratory tests for catecholamine metabolism and tumor marker.

	Patient values	Units	Ref Range	Status
Epinephrine	0.05	nmol/L	<0.34	–
Norepinephrine	0.56	nmol/L	<5.17	–
Dopamine	0.05	nmol/L	<0.31	–
Methoxypinephrine	0.13	nmol/L	<0.42	–
Methoxy norepinephrine	0.37	nmol/L	<0.71	–
3-Methoxytyramine	4.43	pg/ml	<18.40	–
AFP	1.26	ng/ml	<7	–
CA-125	11.38	U/ml	<24	–
CA-199	16.00	U/ml	<30	–
CEA	0.69	ng/ml	<5	–

**Figure 1:**
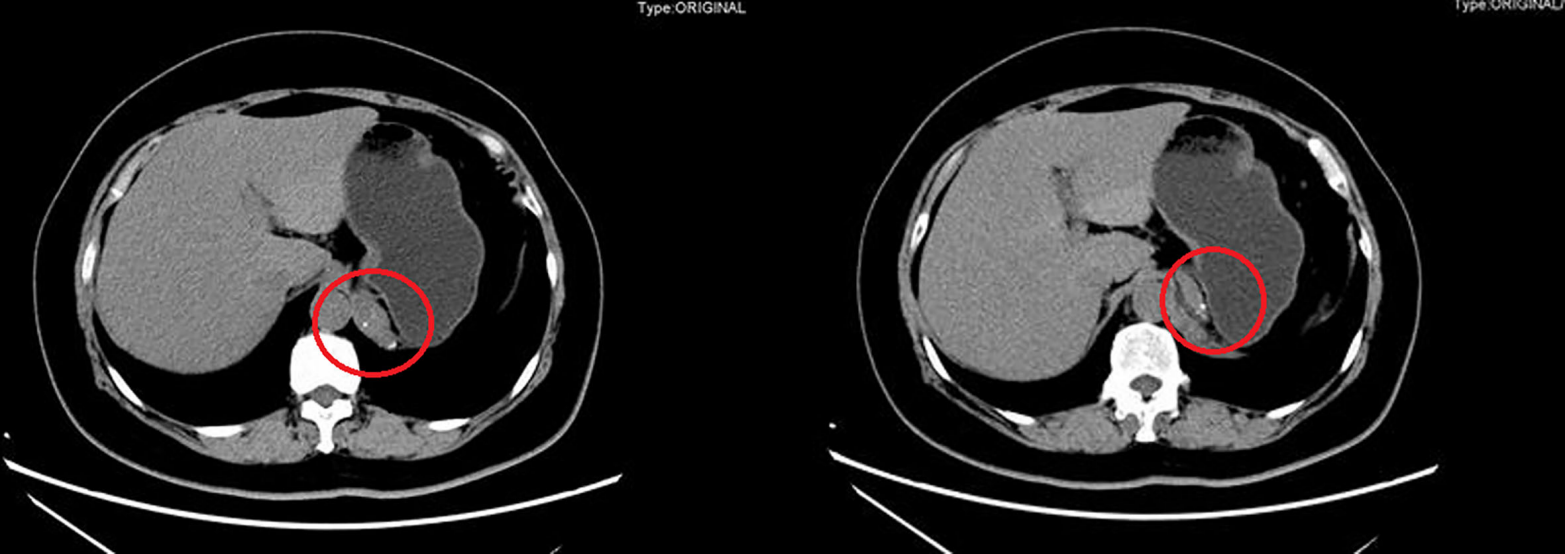
Computed tomography scan showed a 2.2×5.8 cm thin-walled water-attenuated cystic lesion in retroperitoneal region (red circle).

CT showed an ovoid, well-defined, and heterogeneous lesion, measuring 2.2×5.8 cm in her left adrenal area (**Figure 1**). Based on these CT imaging characteristics, a benign lesion (most likely a cyst) was suspected. To confirm the diagnosis and determine the feature, the cyst was completely removed through the laparoscope.

During the surgery, the patient was positioned supine. Using Veress needles, a 1.5-cm incision near the upper navel edge was made to establish pneumoperitoneum with a constant pressure of 13 mm Hg. Four trocars were used: a 5 mm trocar was inserted on the left side of the umbilicus, and two 12 mm trocars were placed below the right costal margin in the midclavicular line and mid-axillary line, respectively. The rest 12 mm trocar was installed under the xiphoid process. The procedure was performed using an ultrasonic surgical aspirator (CUSA; Cavitron Lasersonic Corp., Stamford, CT, USA), an ultrasonic system (Harmonic^®^ scalpel; Ethicon Endo-Surgery, Inc., Cincinnati, OH, USA), and a bipolar clamp coagulation system (ERBE, Tubingen, Germany). Dissociation and exposure of the mass were achieved through the ultrasound knife, along with clamping the blood vessels leading to and from the tumor by a titanium clip. Intraoperatively, a 4×3cm cyst mass was observed behind the head of the pancreas and on the left side of the abdominal aorta, which had an unclear boundary with the surrounding tissues and was closely adhered to the diaphragm. The entire mass was completely removed. Afterward, we repaired the damaged diaphragm and performed complete hemostasis of the wound. The resection specimen was collected in a plastic bag and removed *via* small incisions around the umbilical cord. An orthopedic drainage tube was inserted into the left retroperitoneal cavity, and slightly bloody fluid was collected. The operation lasted 200 min, and the estimated blood loss was 20 ml with no transfusion.

The postoperative period was uneventful and the patient was discharged on the 6th day after surgery with no complications. CT was repeated regularly after surgery. No recurrence, metastasis, or other complications were observed after one and a half years of follow-up.

**Figure 2:**
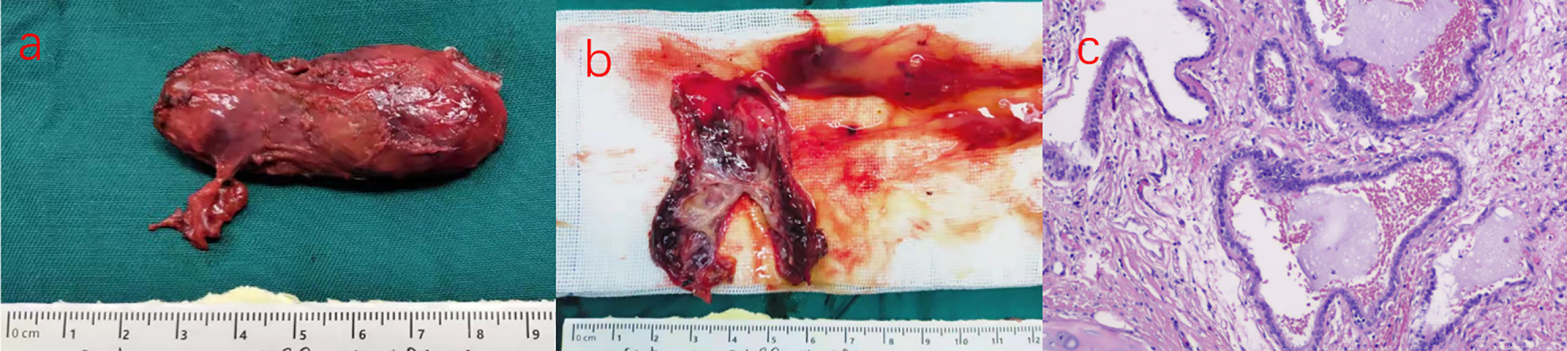
**(A)** An ovoid, well-defined, and homogeneous cystic lesion. **(B)** Mucous filled the cystic lesion. **(C)** Histopathologic section revealed the cyst lined with tall columnar epithelium and cyst wall containing thin smooth muscle bundles, seromucous glands, and mature hyaline cartilage. (Haematoxylin and eosin stain, original magnification, ×200).

Grossly, the cystic lesion measured 60 mm in diameter (**Figure 2A**). Sectioning revealed yellowish fluid within the white tough tissue. Cystic walls were about 1-2 mm thick with a smooth interior surface (**Figure 2B**). Pathologically, characteristic pseudostratified columnar epithelium and cartilage were identified in histological specimens (**Figure 2C**). It was definitively determined that a retroperitoneal bronchogenic cyst existed.

## Discussion

BCs are rare cystic lesions, with the prevalence in the general population remaining unknown (11). Men are slightly more prone to them, and they often go undetected until their 30 s or 40 s (12). They originate predominantly in the middle mediastinum and account for 10% to 15% of mediastinal tumors (13). Occasionally, they are identified in the neck, spinal canal, pleural cavity, skin, esophagus, pericardium, and retroperitoneum (1).

BCs originate from an aberrant budding of the tracheobronchial anlage of the primitive foregut between the 3^rd^ and 7^th^ weeks of embryonic development. The pleuroperitoneal membranes completely seal off the pericardioperitoneal canal at the end of the 7^th^ week. Therefore, the abnormal lung buds are pinched off from the tracheobronchial tree by the growing diaphragm and trapped in the abdominal cavity. Eventually, the early lung buds develop into RBCs (14).

First reported by Miller et al. in 1953, RBCs are extremely rare. A thorough search of the PubMed database revealed 88 publications of retroperitoneal bronchogenic cysts reported worldwide in the English literature between 1991-2022. After screening the full texts and pathological results,40 publications reporting on 45 cases with laparoscopic RBC removal were reviewed within the study. A concise summary of the included articles was shown in **Table 2**.

**Table:** Table 2 Literature review of retroperitoneal bronchogenic cyst undergoing laparoscopic surgery.

	First Author	Year	Country	Sex	Age	Size (cm)	Location	Operation approach	Chief complaint
1	Hu BY (15)	2022	China	M	6	4.5×2.8×8	Left adrenal gland	Laparoscopic	Abdominal pain
2	Hu BY (15)	2022	China	M	18	7.1×3.6×7	Left adrenal gland	Laparoscopic	Asymptomatic
3	Hu BY (15)	2022	China	M	27	3.6×3.5×3.4	Right adrenal gland	Laparoscopic	Asymptomatic
4	Tadokoro T (16)	2022	Japan	F	16	3.8	Under the left diaphragm	Laparoscopic	Upper abdominal pain
5	Cowan S (17)	2021	New Zealand	M	39	3	Left adrenal gland	Laparoscopic	Left flank pain
6	Yuan K (18)	2021	China	F	53	3.3×2.7×3.5	Left adrenal gland	Laparoscopic	Back pain
7	Wu LD (2)	2021	China	F	17	2.9×1.7×2.8	Left adrenal gland	Laparoscopic	Epigastric pain
8	Qingyu J (19)	2021	China	F	41	3.5×3	Left adrenal gland	Laparoscopic	Lumbar back discomfort
9	Wen Y (20)	2020	China	M	27	2.1×4.1	Left adrenal gland	Laparoscopic	Asymptomatic
10	Wen Y (20)	2020	China	M	33	3.1×5.9	Right adrenal gland	Laparoscopic	Asymptomatic
11	Sinha V (21)	2020	India	M	30	7×5	Left adrenal gland	Laparoscopic	Upper abdominal pain
12	Başoğlu M (3)	2018	Turkey	F	38	NA	Left adrenal gland	Laparoscopic	Left upper abdominal pain
13	Liu Q (22)	2018	China	M	33	4.5	Left hepatic hilum	Robotic	Asymptomatic
14	Liu Q (22)	2018	China	M	78	7	Inferior of the left renal vein, left side of IVC	Robotic	Asymptomatic
15	Wang M (8)	2017	China	F	48	8×6×5.5	Left adrenal gland	Laparoscopic	Epigastric pain
16	Yoon YR (23)	2015	Korea	M	57	4.8×2.5	Left adrenal gland	Laparoscopic	Asymptomatic
17	Bulut G (24)	2015	Turkey	F	25	4	Left adrenal gland	Laparoscopic	Left flank pain
18	Jiang X (25)	2015	China	M	52	2.5×2.5×0.5	Left crus of the diaphragm	Laparoscopic	Asymptomatic
19	Trehan M (26)	2015	India	F	34	10×6	Right hypochondrium	Laparoscopic	Right flank heavy
20	Zhang D (27)	2015	China	M	8	4	Left adrenal gland	Laparoscopic	Asymptomatic
21	Terasaka T (28)	2014	Japan	M	27	5.4×3.8	Left adrenal gland	Laparoscopic	Asymptomatic
22	Cao DH (29)	2014	China	M	51	4.5	Left adrenal gland	Laparoscopic	Headache
23	Dong B (30)	2014	China	F	30	1.5×2×2	Left adrenal gland	Laparoscopic	Asymptomatic
24	Castro R (31)	2013	Portugal	F	36	8	Left upper quadrant	Laparoscopic	Abdominal pain
25	Runge T (32)	2013	Switzerland	F	42	5×3.6×4	Left adrenal gland	Laparoscopic	Epigastric pain
26	Cai Y (33)	2013	China	F	50	3	Pancreas posterior wall	Laparoscopic	Left flank pain
27	Jannasch O (34)	2013	Germany	M	50	4	Left adrenal gland	Laparoscopic	Left flank pain
28	O'Neal PB (35)	2012	USA	F	23	5.2×4	Left adrenal gland	Laparoscopic	Abdominal discomfort
29	Alguraan Z (36)	2012	USA	F	23	4	Right adrenal gland	Robotic	Asymptomatic
30	Díaz Nieto R (37)	2010	Spain	M	67	6	Gastroesophageal junction	Laparoscopic	Low back pain
31	Inaba K (38)	2010	Japan	F	64	3×4×2	Stomach posterior wall	Laparoscopic	Asymptomatic
32	El Youssef R (39)	2010	Portland	M	44	3	Left adrenal gland	Laparoscopic	Asymptomatic
33	Obando J (40)	2009	USA	M	67	3.9×3.7	Left upper-quadrant	Laparoscopic	Asymptomatic
34	Chung JM (41)	2009	Korea	F	41	4.8×3.5×4.2	Left adrenal gland	Laparoscopic	Asymptomatic
35	Roma A (42)	2008	USA	M	40	6.2	Left adrenal gland	Laparoscopic	Asymptomatic
36	Minei S (43)	2007	Japan	M	39	3.5×3	Left adrenal gland	Laparoscopic	Fever
37	Chu PY (44)	2007	China	M	55	4×3	Left adrenal gland	Laparoscopic	Asymptomatic
38	Terry NE (45)	2007	USA	F	75	5	Left adrenal gland	Laparoscopic	Abdominal pain
39	Ishizuka O (46)	2004	Japan	M	36	5×3	Left adrenal gland	Laparoscopic	Asymptomatic
40	Ishikawa T (47)	2003	Japan	F	41	9.2	Left adrenal gland	Laparoscopic	Left flank pain
41	Hedayati N (48)	2003	USA	F	59	7×5	Left adrenal gland	Laparoscopic	Asymptomatic(convert to open )
42	McCrystal DJ (49)	2002	Australia	F	8	4×3×2	Left adrenal gland	Laparoscopic	Abdominal pain
43	McCrystal DJ (49)	2002	Australia	M	15	5.5×3.5×1.2	Left adrenal gland	Laparoscopic	Left flank pain
44	Yamamoto E (50)	1998	Japan	F	49	3.2×2.2	Right adrenal gland	Laparoscopic	Asymptomatic
45	Tokuda N (51)	1997	Japan	F	24	3	Left adrenal gland	Laparoscopic	Asymptomatic

Laparoscopic resection was first reported in 1997 by Tokuda et al. for 3 cm such cysts (51). This procedure of the cyst has been reported most frequently in China (17cases, 37.8%), followed by Japan (8 cases,17.8%) and the United States (6 cases,13.3%). The finding concerning regional and race differences was consistent with Mike et al. (52) However, it remained unclear whether this represents a real difference in incidence among Asian patients or merely a reporting bias. There exists no discrepancy between the gender who underwent laparoscopic surgery (22 female and 23 male), with an average diagnosis age of 38.6 (range 6-78) years. To date, the largest retroperitoneal bronchial cyst of laparoscopic excision was 10×6cm, as reported by Trehan et al. in 2015 in India (26). Retroperitoneal bronchogenic cysts tend to be found on the left side of the abdomen(37cases,82.2%). The most common location of retroperitoneal bronchogenic cyst is near the left adrenal gland (31cases, 68.9%). Only 4 cases (8.9%) were discovered in the right adrenal gland. The previous review of cases has confirmed this difference (18). Based on Rud et al. (53), the left pericardioperitoneal canal closes later and is larger than the right which can explain why RBCs prefer to be located on the left side. It’s worth noting that nearly half of patients (22cases, 48.9%) find a mass incidentally, with no typical clinical manifestations. Of symptomatic patients, the majority complained of abdominal pain (7 cases, 30.4%) left flank pain (6 cases,26.1%) and a small number complained of thoracic pain and back pain.

Similar literature reviews were successively performed by Cetinkurşun et al. (54), Mike et al. (52), Govaerts et al. (55), and Yuan et al. (18) in 1997, 2005, 2012, and 2021, respectively. The aforementioned studies, however, included a large number of patients undergoing surgery *via* the open approach. In addition, we enrolled the latest reports from the past two years through a more comprehensive search, and supplemented cases missed by the previous retrieval. Similar conclusions were obtained regarding the clinical characteristics of the disease, including age, symptoms, and predilection sites.

It remains difficult to diagnose RBCs before surgery. CT and MRI are the most helpful imaging modalities (13, 56). Typical CT appearances are sharply defined, homogeneous masses with attenuation coefficients to water density (0 to 20 HU). However, the attenuation coefficients can increase when protein, calcium, or anthracosis pigment are elevated within the cyst, aggravating difficulties in the differential diagnosis (14). MRI reveals the inhomogeneity of RBCs with better clarity than CT, providing a more accurate preoperative diagnosis (56). By now, only histopathology can yield a definitive diagnosis of BCs. Indispensable pathological criteria consist of secretory epithelium along with bronchial glands, smooth muscle, or hyaline cartilage (57). There is still no exact evidence that endocrine or tumor biomarkers are associated with RBCs. In only a few cases were endocrine or tumor biomarker variations recorded (8, 58, 59).

Early surgical excision of RBCs is recommended to clarify a diagnosis, relieve symptoms and prevent complications, even if asymptomatic. Due to reduced operating time, length of stay, and intraoperative blood loss, laparoscope has been increasingly ubiquitous in intraperitoneal surgery. Currently, laparoscopic resection is particularly recommended to clarify the diagnosis and apply for the treatment of retroperitoneal bronchogenic cysts. In addition, laparoscopic resection has been widely used to lessen the economic burden as well as postoperative pain of patients. More than half of patients with RBCs underwent laparoscopic resection and the majority were free of complications during the postoperative course. The present study also found laparoscopy to be safe and reliable. In particular, the retroperitoneal laparoscopic excision, which was first documented by McCrystal et al. in 2002, has gained more and more applications in recent years. Our literature review supports that the retroperitoneal approach is feasible, effective, and less invasive when treating such retroperitoneal cysts. In addition, there are only two articles reporting three cases of robotic surgery applicated to RBC patients (22, 36). Robotic surgery has overcome many limitations of traditional laparoscopic surgery and has improved in terms of dexterity, tremor reduction, and 3-dimensional visualization (60). This enables broader adaptability of robotic surgery in abdominal and retroperitoneal surgical procedures (61). However, due to the high financial cost, the application of robotic resection remains limited (22).

The prognosis of bronchogenic cysts after surgical excision is excellent. No recurrence, malignancy, or other complications are reported in all the literature we reviewed.

To our best knowledge, our review provides the largest case series in the world with such cysts to be resected laparoscopically. In conclusion, we reported a case with an ectopic bronchogenic cyst in the left retroperitoneal region. A literature review suggests that laparoscopic excision is optimal management to establish both diagnosis and treatment. The long-term outcome of this disease is excellent, with no report of recurrence.

## Data availability statement

The original contributions presented in the study are included in the article/supplementary material. Further inquiries can be directed to the corresponding author.

## Ethics statement

Written informed consent was obtained from the individual(s) for the publication of any potentially identifiable images or data included in this article.

## Author contributions

JL and QF contributed to the study concept and design. HL, JX, and QF contributed to the investigation and writing the original draft. HL, JX, and QF contributed to data collection. QF,JL and ZC revised the paper. All authors contributed to the article and approved the submitted version.

## Funding

This work was supported by the 2021 Sichuan Science and Technology Plan Project “International cooperation in science and technology innovation/technological innovation cooperation in Hong Kong, Macao and Taiwan“ (2021YFH0095) and Sichuan University from 0 to 1 project (No. 2022SCUH0017).

## Conflict of interest

The authors declare that the research was conducted in the absence of any commercial or financial relationships that could be construed as a potential conflict of interest.

## Publisher’s note

All claims expressed in this article are solely those of the authors and do not necessarily represent those of their affiliated organizations, or those of the publisher, the editors and the reviewers. Any product that may be evaluated in this article, or claim that may be made by its manufacturer, is not guaranteed or endorsed by the publisher.
